# MiR-128-3p Alleviates Spinal Cord Ischemia/Reperfusion Injury Associated Neuroinflammation and Cellular Apoptosis via SP1 Suppression in Rat

**DOI:** 10.3389/fnins.2020.609613

**Published:** 2020-12-23

**Authors:** Dan Wang, Fengshou Chen, Bo Fang, Zaili Zhang, Yan Dong, Xiangyi Tong, Hong Ma

**Affiliations:** Department of Anesthesiology, The First Hospital of China Medical University, Shenyang, China

**Keywords:** spinal cord ischemia/reperfusion injury, miR-128-3p, SP1, neuroinflammation, apoptosis, rat

## Abstract

**Background:**

Neuroinflammation and cellular apoptosis caused by spinal cord ischemia/reperfusion (I/R) injury result in neurological dysfunction. MicroRNAs (miRs) have crucial functions in spinal cord I/R injury pathogenesis according to previous evidences. Herein, whether miR-128-3p contributes to spinal cord I/R injury by regulating specificity protein 1 (SP1) was assessed.

**Methods:**

A rat model of spinal cord I/R injury was established by occluding the aortic arch for 14 min. Then, miR-128-3p’s interaction with SP1 was detected by dual-luciferase reporter assays. Next, miR-128-3p mimic and inhibitor, as well as adenovirus-delivered shRNA specific for SP1 were injected intrathecally for assessing the effects of miR-128-3p and SP1 on rats with spinal cord I/R injury. SP1, Bax and Bcl-2 expression levels in I/R injured spinal cord tissues were evaluated by Western blotting, while IL-1β, TNF-α, and IL-6 were quantitated by ELISA. Tarlov scores were obtained to detect hind-limb motor function. Evans blue (EB) dye extravasation was utilized to examine blood–spinal cord barrier (BSCB) permeability. Terminal deoxynucleotidyl transferase mediated dUTP nick end labeling (TUNEL) staining was performed for neuronal apoptosis assessment.

**Results:**

MiR-128-3p expression was decreased, while SP1 amounts were increased in rat spinal cord tissue specimens following I/R. SP1 was identified as a miR-128-3p target and downregulated by miR-128-3p. MiR-128-3p overexpression or SP1 silencing alleviated I/R-induced neuroinflammation and cell apoptosis, and improved Tarlov scores, whereas pretreatment with miR-128-3p inhibitor aggravated the above injuries.

**Conclusion:**

Overexpression of miR-128-3p protects neurons from neuroinflammation and apoptosis during spinal cord I/R injury partially by downregulating SP1.

## Introduction

Spinal cord I/R injury represents the most catastrophic complication generally ensuing multiple events, including thoracoabdominal aortic surgery and spinal cord injury (Wong et al., 2011; Etz et al., 2015). Spinal cord I/R injury may eventually lead to high incidence of paralysis or paraplegia (LeMaire et al., 2012; Coselli et al., 2016). Despite its improvement by clinical operative methods and perioperative approaches, spinal cord I/R injury cannot be prevented completely (Saito et al., 2013; Karatas et al., 2019). Owing to complicated pathogenic factors, such as oxygen free radical-associated lipid peroxidation, excessive calcium accumulation within the cell, leukocyte induction, inflammation, and neuronal apoptosis, which are rapidly induced and evoke serious complications (Li et al., 2014c). Therefore, it is essential to investigate the exact mechanism of spinal cord I/R injury and develop effective therapeutic measures.

SP1 belongs to the specificity protein/Kruppel-like factor family that also comprises SP2, SP3, and SP4 (Wierstra, 2008). Emerging evidences reveal SP1 as an important regulator of multiple cell events such as cell cycle, proliferation, and apoptosis (Nam et al., 2014; Beishline and Azizkhan-Clifford, 2015). Citron et al. suggested that SP1 was expressed in cerebral tissues, with elevated amounts decreasing neuronal survival (Citron et al., 2008). In addition, SP1 was shown to be upregulated in the development of neurological pathologies, including PD (Cai et al., 2020; Wang et al., 2020) and Alzheimer’s diseases (Ramanan and Saykin, 2013). Furthermore, Qin et al. (2018) suggested that suppressing SP1 activation effectively inhibited OGD/R-induced inflammatory activation in microglia. Recent evidence indicated that the expression of SP1 under ischemia/hypoxic conditions was increased (Li et al., 2019). Jointly, the aforementioned data revealed the critical influence of SP1 in neurological pathologies.

MicroRNAs (miRNAs) represent a group of small non-coding RNAs with 21–23 nucleotides, which are able to interact with the 3′-UTRs of mRNAs and negatively modulate gene expression via cleavage or translational suppression at the posttranscriptional level (Kim et al., 2009). MiRNAs are highly expressed in the central nervous system, with crucial roles in neuron development, maintenance of neuronal phenotype, and neuroprotection (Saugstad, 2010). In addition, many miRNAs are dysregulated in the spinal cord during the pathogenesis of I/R injury (Song et al., 2016; Balsam, 2017). Meanwhile, synthetic miRNA mimics and anti-miRNA oligonucleotides powerfully mitigate neuroinflammation and neuroapoptosis, and elevate neurological functional recovery in rat models (He et al., 2015; Li et al., 2015; Liu et al., 2017; Li et al., 2018). Recently, miR-128-3p, which is best known as a tumor suppressor (Huang et al., 2015; Zhao et al., 2018), has been found to contribute to central nervous system disorders such as PD (Zhang et al., 2020) and glioma (Huo et al., 2019). Mao et al. (2017) demonstrated miR-128-3p prevented neuron apoptosis by regulating p38α in the initial stage of brain I/R. However, SP1’s role in spinal cord I/R-associated injury and the mechanism by which miR-128-3p presents I/R remain unclear. Therefore, the current work aimed to assess the functions of miR-128-3p and SP1 in spinal cord I/R injury. We hypothesized that miR-128-3p relieves neurological damage in spinal cord I/R injury by depleting SP1 to decrease inflammatory reactions and apoptosis. This pathway could emerge as a possible target relieving spinal cord I/R injury -related nerve injury.

## Materials and Methods

### Animals

In this study, male Sprague-Dawley rats (200–250 g) were provided by the Animal Center of China Medical University, Shenyang (China). All animals (*n* = 136) were housed for at least 1 week before the surgical operation, with freely available rodent chow and water at 22–24°C and 50–60% relative humidity, under a 12 h/12 h light-dark cycle. Animal experiments had approval from the Ethics Review Committee for Animal Experimentation of China Medical University, Shenyang (China) (CMU2020267/2020/03/02), and were carried out in conformity with the National Institutes of Health Guide for the Use and Care of Laboratory Animals (NIH Publications No.80-23, revised 1996).

### Rat Model of Spinal Cord I/R Injury

Spinal cord I/R injury was induced in rats via a cross-clamped aortic arch (Li et al., 2014b,c). Briefly, upon anesthesia by intraperitoneally injecting 4% sodium pentobarbital (50 mg/kg; Beyotime Biotechnology, China), endotracheally intubated (24 g trocar sleeve) and lung ventilation was accomplished utilizing a small-animal ventilator (Harvard Apparatus, United States) (tidal 15 mL/kg, breathing frequency 80–100 times/min, breathing ratio 1:1). Body temperatures were kept at 37.5 ± 0.5°C as monitored with a rectal probe. Under aseptic conditions, the left common carotid artery was exposed in the middle of neck. Then, the aortic arch was uncovered through a cervicothoracic incision. Under direct vision, the aortic arch was cross-clamped for 14 min between the left carotid artery and the left subclavian artery to induce ischemia. A catheter (24 g trocar sleeve) was inserted into the femoral artery for blood pressure measurements. After ischemia confirmation [90% reduction of the flow assessed at the femoral artery using a laser Doppler blood flow monitor (Moor Instruments, Axminster, Devon, United Kingdom)], clamp removal was performed, followed by 12-h reperfusion. This procedure was performed in sham animals, except for blockade.

### Experimental Protocol

**FIGURE 1:**
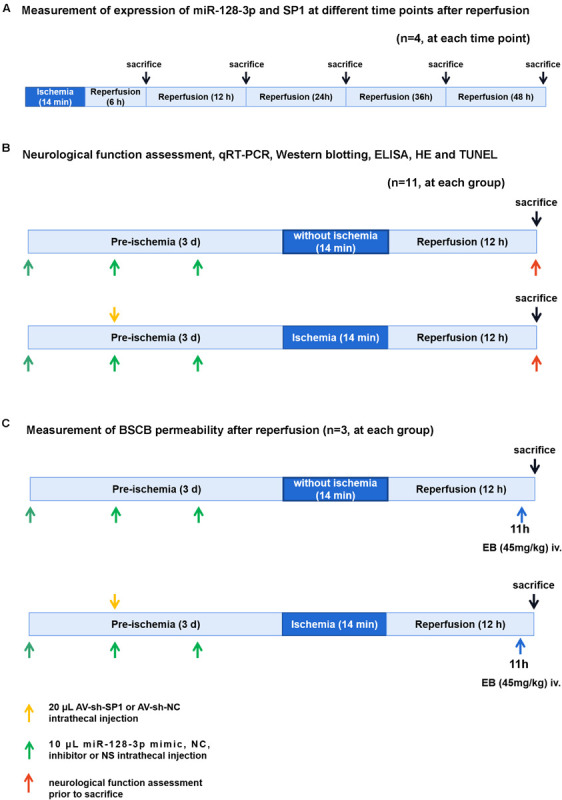
Diagram of the experimental protocols. **(A)** Measurement of expression of miR-128-3p and SP1 at different time points (6, 12, 24, 36, and 48 h) after reperfusion. *n* = 4, at each time point. **(B)** Neurological function assessment (*n* = 8, at each group), qRT-PCR and Western blotting (*n* = 4, at each group), ELISA (*n* = 4, at each group), H&E and TUNEL (*n* = 3, at each group). Rats (*n* = 8, at each group) were euthanized after neurological function assessment. Then 4 rats’ spinal cords were used for qRT-PCR and Western blotting and the other 4 rats’ spinal cords were used for ELISA. **(C)** Measurement of BSCB permeability after reperfusion (*n* = 3, at each group).

Experimental groups and protocol were shown in Figure 1. We used 118 rats and 18 rats for modeling and sham operation, respectively. To measure expression of miR-128-3p and SP1 at various time points (6, 12, 24, 36, and 48 h) after reperfusion, 20 rats were killed at 6, 12, 24, 36, and 48 h after spinal cord I/R, respectively (*n* = 4, at each time point). Subsequently, eight groups were enrolled: (1) Sham group (*n* = 14): same procedure was performed on rats without blockade and 10 μL normal saline was injected inreathecally into each rat prior to sham-operation. (2) I/R group (*n* = 14): cross-clamping for 14 min on rats for ischemia induction. (3) miR-128-3p mimic group (*n* = 14): 10 μL synthetic miR-128-3p mimics were injected intrathecally into each rat prior to ischemia induction once a day for three consecutive days. (4) miR-128-3p negative control (NC) group (*n* = 14): 10 μL synthetic miR-128-3p NC was injected intrathecally into each rat prior to ischemia induction once a day for three consecutive days. (5) miR-128-3p inhibitor group (*n* = 14): 10 μL synthetic miR-128-3p inhibitors were injected intrathecally into each rat prior to ischemia induction once a day for three consecutive days. (6) Adenovirus (AV)-sh-SP1 group (*n* = 14): 20 μL shRNA-SP1 adenovirus at 5 × 10^10^ PFU/mL was carried out for 2 days before ischemia. (7) AV-sh-NC group (*n* = 14): 20 μL shRNA-NC adenovirus at 5 × 10^10^ PFU/mL was carried out for 2 days before ischemia. (8) AV-sh-SP1 + miR-128-3p inhibitor group (*n* = 14): 10 μL synthetic miR-128-3p inhibitor were injected intrathecally into each rat prior to ischemia induction once a day for three consecutive days and 2 days before ischemia 20 μL shRNA-SP1 adenovirus at 5 × 10^10^ PFU/mL was carried out.

### Intrathecal Administration

Intrathecal administration of various substances followed a previously reported method (Mestre et al., 1994; Li et al., 2015; He et al., 2016; Li et al., 2018). In brief, upon anesthesia with isoflurane (RWD, Shenzhen, China), rats were placed in the prone position that flexed the rats’ back. A 20-μL microsyringe (Gaoge Co., Ltd., Shanghai, China) was positioned in the subarachnoid space at the intervertebral space between L5 and L6 or L4 and L5. The spinal cord of rat terminated between the third and fourth lumbar vertebrae (Padmanabhan and Singh, 1979). This site was chosen so that the injection was restricted to the region where the spinal cord ends and the cauda equine begins in order to minimize the possibility of spinal damage and maximize intervertebral accessibility (Mestre et al., 1994). According to the anatomical structure of the rat, the horizontal position of the connection between the left and right hip joints corresponds to the spinous process space of L5–L6 or the spinous process of L5. When the needle entered the subarachnoid space, a sudden lateral tail-flick was used as a sign of successful puncture. Once correct needle placement was confirmed, the solutions were injected slowly and over the course of 1 min (Judas et al., 2014). Synthetic miR-128-3p mimic, inhibitor and negative control were purchased from Transheep (Shanghai China). A shRNA-SP1 adenovirus (AV-sh-SP1) and NC adenovirus (AV-sh-NC) were provided by IGE Bio. Ltd., (Guangzhou, China). Rats were intrathecally infused with 10 μL of synthetic miR-128-3p mimic, inhibitor and NC, respectively, at a concentration of 500 pmol/10 μL with Entranster^TM^-*in vivo* transfection regent (Engreen, Beijing, China) to regulate miR-128-3p *in vivo* prior to ischemia induction once a day for three consecutive days. Meanwhile, 2 days before ischemia, intrathecal infusion of 20 μL of shRNA-SP1 or NC adenovirus at 5 × 10^10^ PFU/mL was carried out, as directed by the manufacturer. Transfection efficacy was assessed by qRT-PCR or Western blotting. Only animals with unaltered motor function after intrathecal injection underwent surgery. The sequences of miR-128-3p mimic, miR-128-3p inhibitor, miR-128-3p NC and AV-sh-SP1 were as follows: miR-128-3p mimic (5′-UCACAGUGAACCGGUCUCUUU-3′), miR-128-3p inhibitor (5′-AGUGUCACUUGGCCAGAGAAA-3′), miR-128-3p NC (5′-CAGUACUUUUGUGUAGUAAA-3′), AV-sh-SP1 (5′-CCGGCCAACTTACAGAACCAGCAAGTTCTCTCGAGAGAA CTTGCTGGTTCTGTAAGTTGGTTTTTGAATT-3′).

### Neurological Assessment

Hind-limb motor function was examined at 12 h post reperfusion (*n* = 8, at each group) by two investigators blinded to experimental design according to the Tarlov scoring system (Aslan et al., 2009; Li et al., 2014a): 0, Spastic paraplegia and no voluntary movement of lower limbs; 1, Spastic paraplegia and weak hind limb motor function of lower limbs; 2, good antigravity strength with some lower limb movement, but inability to stand; 3, abnormal standing but unable to walk normally; 4, normal motor function.

After neurological evaluation, the rats in all groups were anesthetized with 4% sodium pentobarbital 50mg/kg and perfused transcardially with normal saline. The spinal cords from L4 to L6 were harvested for molecular biology analysis because of their vulnerability to ischemic injury (Li et al., 2014a). There are two methods to identify the L4–L6 segments. On the one hand, in the rat, the sciatic nerve is composed of spinal segments L4, 5, and 6. The roots of these three spinal nerves originate in the spinal cord within the limits of the L1 vertebra, and then must descend distances of 2.7 cm (L4) to 4.5 cm (L6) within the vertebral canal before reaching the periphery (Gelderd and Chopin, 1977). Therefore, L4–L6 spinal segments were identified based on the ascending ends of the posterior fibers of the dorsal root ganglion of L4, 5, and 6. On the other hand, according to the anatomical morphology of the rat spinal cord, the lumbar enlargement of spinal cord in rat is located at the level of vertebrae T12-L1, which corresponds to spinal cord segments L1–L5 (Gilerovich et al., 2008). The L4–L6 segments lie at the upper border, middle and lower border of the L1 vertebra. Therefore, L4–L6 spinal cord segments were identified based on the position of L1 vertebra. At the end of these procedures, all rats were sacrificed under deep anesthesia.

### Quantitative Real-Time Polymerase Chain Reaction (qRT-PCR)

After final neurological assessment, total RNA from L4–L6 spinal cord specimens (*n* = 4, at each group) was obtained with TRIzol reagent (Takara, Japan) as instructed by the manufacturer. Then, reverse transcription utilized Prime-Script RT reagent Kit with gDNA Eraser (TaKaRa) or MicroRNA Reverse Transcription Kit (Applied Biosystems, United States). Subsequently, qRT-PCR was performed with a SYBR PremixEx TaqII kit (Takara) on an ABI 7500 qRT-PCR instrument (Applied Biosystems) at 95°C (10 min), followed by 40 cycles at 95°C (20 s), 60°C (60 s), 62°C (30 s), and 72°C (30 s). Relative SP1 mRNA and miR-128-3p amounts were assessed by the 2^–ΔΔ*Ct*^ method, with GAPDH and U6 as internal references, respectively. The primers (Sangon Biotech, China) employed were as follows: miR-128-3p (forward: 5′-CGTCACAGTGAACCGGTCTCTTT-3′), SP1 (forward: 5′-CTGCAAGGGTCTGATTCTCTA-3′, reverse: 5′-AGCTTGTCCACCTTGAACTA-3′), GAPDH (forward: 5′-GGGGCTCTCTGCTCCTCCCTG-3′, reverse: 5′-AGGCGTCC GATCGGCCAAA-3′), U6 (forward: 5′-CTCGCTTCGGCAGC ACA-3′, reverse: 5′-AACGCTTCACGAATTTGCGT-3′).

### Cell Culture

293T cell lines were purchased from cell bank center of Shanghai Institutes for Biological Sciences (SIBS) and were cultured in DMEM (Invitrogen, Thermo Fisher Scientific, Waltham, MA, United States) appended with 10% FBS and 2 Mm glutamine, and 100 μg/ml streotonucin and penicillin (Sangon Technology, Shanghai, China). All of the cells were incubated at 37°C with 5% CO_2_ (Yang et al., 2019).

### Dual Luciferase Reporter Gene Assay

MiR-128-3p target prediction was performed with TargetScan^1^. For constructing 3′-UTR-luciferase reporters, regions of SP1’s 3′-UTR were introduced into pmirGLO (Promega). Then, two wild types named WT1-3′ UTR (binding sites at base pairs [bp] 1437–1443 and 1642–1649) and WT2-3′ UTR (binding site at bp 4373–4379) were constructed by Nanjing TsingKe Co., Ltd. (China). Mutant-type plasmids (MUT1-3′ UTR and MUT2-3′ UTR) were generated with the Directed Mutagenesis system (Invitrogen). Meanwhile, SP1 WT and MT plasmids, respectively, were co-transfected with miR-128-3p mimic or miR-128-3p -NC in 293 T cells with Lipofectamine 2000 (Invitrogen) as directed by the manufacturer. After transfection for 48 h, cell collection and homogenization in passive lysis buffer (Promega G182A) (100 μl) were carried out. Renilla and firefly luciferase activities in triplicate assays were assessed with Luciferase Assay Kit (Beyotime) on a multifunctional microplate reader (PerkinElmer EnSpire, United States).

### Hematoxylin and Eosin (H&E) Staining

At 12 h post-modeling, rats (*n* = 3, at each group) were deeply anesthetized with 4% sodium pentobarbital (50 mg/kg, i.p.). Then, they were perfused transcardially with normal saline, followed by 4% buffered paraformaldehyde (Servicebio G1101, China), and until the right atrium flowed out clear paraformaldehyde and the lungs turned white (Li et al., 2019; Yin et al., 2019). The spinal cords from L4 to L6 were removed rapidly for analysis because of their vulnerability to ischemic injury (Li et al., 2014a). Tissue was fixed with 4% paraformaldehyde for 24 h and embedded in paraffin. Then, paraffin-embedded sections (4 μm) underwent staining with hematoxylin and eosin (H&E) staining kit (Servicebio G1005, China) as directed by the manufacture. Briefly, the 4 μm thick paraffin-embedded sections were firstly deparaffinized and hydrated to water. Second, the sections were stained in hematoxylin solution for 5 min and rinsed in water. Then, the sections were differentiated in 1% hydrochloric acid ethanol solution and restored blue in ammonia solution and washed in slowly running tap water. After then, the sections were dehydrated with 85 and 95% gradient alcohol III, Xylene I, and Xylene II for 5 min, respectively. Stained sections were examined under an optical microscope (Nikon Eclipse E100, Japan) by two blind investigators to judge the morphological appearance of neurons. Normal neurons were defined as viable by the existence of basophilic stippling, whereas necrotic or dead neurons were identified by the existence of diffusely eosinophilic cytoplasm with pyknotic homogenous nuclei (Ding et al., 2009; Li et al., 2014a). Gray matter injury was examined by counting normal motor neurons in its ventral region. Data were calculated as average numbers of intact neurons using ImageJ software (Download from the NIH website^2^) (Li et al., 2014a). Three sections for each rat were assessed in a blinded fashion.

### Terminal Deoxynucleotidyl Transferase dUTP Nick End Labeling (TUNEL)

The TUNEL assay was extensively carried out for the detection and quantification of apoptosis in histological tissue sections (Duan et al., 2003). The above tissue sections (*n* = 3, at each group) underwent staining with TMR (red) TUNEL Cell Apoptosis Detection Kit (Servicebio, G1502, China) as directed by the manufacturer. Following TUNEL labeling, cell nuclei were stained with DAPI (Servicebio G1012, China) for 5 min. Cell apoptosis was assessed by two blinded investigators under a light microscope (Leica, Germany). The nuclei labeled with DAPI appear blue under a fluorescence microscope and the positive apoptotic nuclei were red. The TUNEL-positive cells were recognized as cells double labeled with TUNEL and DAPI and only TUNEL + DAPI were counted. (Li et al., 2018). Finally, the high power fields of spinal cord were randomly selected, and average percentage in three sections/rat of TUNEL-positive cells to total nuclei were calculated by two blind investigators using ImageJ software (Download from the NIH website^2^). The apoptotic rate was calculated as AR = (number of TUNEL-positive cells/total number of nuclei) × 100% (Fu et al., 2020).

### Evans Blue (EB) Extravasation

Evans blue (EB) extravasation was carried out to quantitatively and qualitatively assess blood–spinal cord barrier (BSCB) integrity following I/R-associated injury (*n* = 3, at each group) (Li et al., 2015). After 12 h of reperfusion, Evans blue (EB, 1 mg/ml; Solarbio, China) was administered by intravenous (tail vein) injection (45 mg/kg) 60 min before euthanasia. The rats were anesthetized and perfused transcardially with 500 mL/kg normal saline, and the L4–L6 segments were removed and underwent fixation with 4% formalin and sectioning at 10 μm (Fang et al., 2013b). EB staining was evaluated under a fluorescence microscope (Olympus, Melville, NY, United States).

### Enzyme Linked Immunosorbent Assay (ELISA)

Following 12 h of modeling, L4–L6 segments were obtained (*n* = 4, at each group), homogenized in chilled PBS and submitted to centrifugation (3,000 r/min, 15 min; 4°C). Then, interleukin (IL)-1β, IL-6 and TNF-α amounts were quantified on a microplate reader with ELISA kits (MultiSciences Biotech Co., Ltd., China), as described by the manufacturer. Triplicate experiments were assessed at 450 nm, and results were normalized to protein amounts.

### Western Blotting

L4–L6 segments (*n* = 4, at each group) underwent lysis with radioimmunoprecipitation (RIPA) lysis buffer (R0010, Solarbio) supplemented with PMSF (P0100, Solarbio) as previously described (Li et al., 2018). Then, protein concentrations were quantified with a BCA protein assay kit (Beyotime Biotechnology). Equal amounts of protein were resolved by SDS-PAGE (KGP113K, KeyGen Biotech. Co. Ltd., Nanjing, China) and electro-transferred onto PVDF membranes (Millipore, Temecula, CA, United States). Upon blocking with 5% non-fat milk (1 h), the membranes underwent overnight incubation with anti-SP1 (Abcam, ab13370; 1:1000), anti-Bax (Cell Signaling Technology, 2772; 1:1000), anti-Bcl-2 (ProteinTech, 26593-1-AP; 1:1000) and β-actin (ZSGB-BIO, China; 1:5000) primary antibodies at 4°C. Next, the specimens were incubated with HRP-conjugated goat anti-rabbit (ProteinTech, SA00001-2) or anti-mouse (ProteinTech, SA00001-1) antibodies (1 h, 37°C). The membranes were further washed with TBST 3 times. An ECL kit (Bio-Rad, United States) was utilized for visualization, and bands were detected by the Quantity One software (Bio-Rad). ImageJ 1.6.0.24 (National Institutes of Health, United States) was used to quantify the band strength; β-actin served as a reference protein.

### Statistical Analysis

SPSS 25.0 (SPSS, United States) was utilized for data analysis. Data are mean ± SD. The Student’s *t*-test or Mann–Whitney test was performed for group pair comparisons, while multiple groups were assessed by one-way or two-way ANOVA, with *post hoc* Tukey test. *P* < 0.05 was deemed statistically significant.

## Results

### Abnormal Expressions of miR-128-3p and SP1 After I/R

**FIGURE 2:**
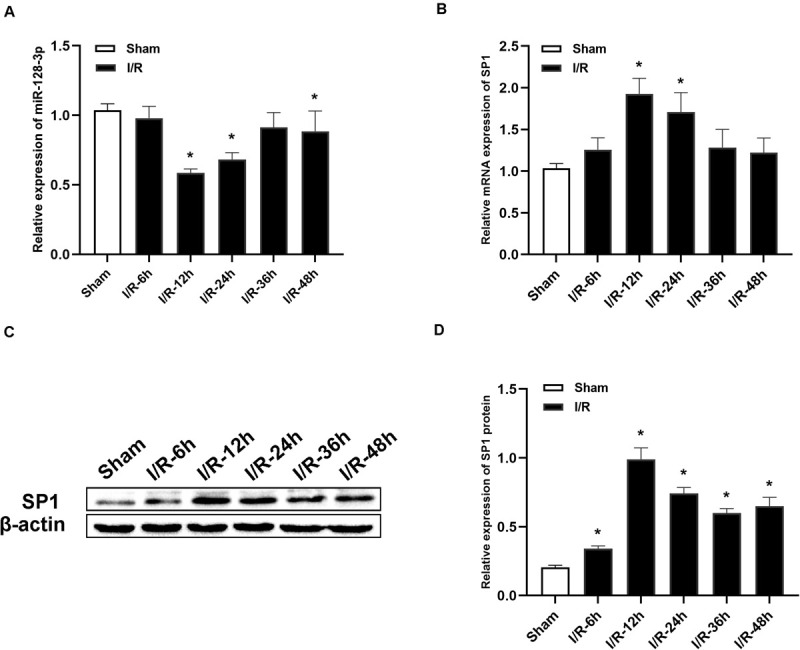
Alterations in miR-128-3p and SP1 expression levels at various time points after spinal cord I/R injury. **(A)** Quantification of miR-128-3p expression in the L4–L6 segments of spinal cord specimens after spinal cord I/R injury. **(B)** Quantification of SP1 mRNA expression in the L4–L6 segments of spinal cord specimens after spinal cord I/R injury. **(C,D)** Western blotting analysis of SP1 protein levels in the L4–L6 segments of spinal cord specimens after spinal cord I/R injury. β-actin was used as a loading control. One-way ANOVA was performed for multiple- group comparisons, followed by the Tukey’s test. *P < 0.05 vs. sham group.

MiR-128-3p and SP1 amounts were assessed at 6, 12, 24, 36, and 48 h post-I/R, respectively. As shown in Figure 2A, qRT-PCR revealed that miR-128-3p expression was noticeably diminished over time and reached its minimum amounts at 12 h after I/R, in comparison with the sham group (*P* < 0.05). Additionally, qRT-PCR and Western blotting analysis revealed that SP1 mRNA and protein levels were elevated from 6 h post-I/R in comparison with the sham group, and peaked at 12 h (Figures 2B–D, *P* < 0.05).

### SP1 Is a Direct miR-128-3p Arget

**FIGURE 3:**
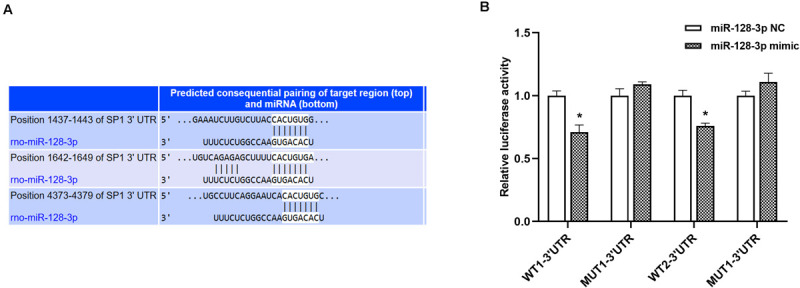
SP1 is a direct miR-128-3p target. **(A)** Prediction of miR-128-3p and SP1 binding sites based on TargetScan. Three predicted conserved miR-128-3p target sites in the 3′UTR of SP1. **(B)** Suppression of SP1 expression by miR-128-3p. Luciferase activity was measured after 48 h of co-transfection of 293T cells with the miR-128-3p mimic and the WT or MUT reporter vector. Compare to the control, miR-128-3p significantly decreased the luciferase activity of the WT vector but not that of the MUT vector. 3′ UTR, 3′untranslated region; WT, wild-type; MUT, mutant-type, SP1, specificity protein 1; NC, negative control. *P < 0.05 vs. NC group.

According to results from TargetScan database, a putative miR-128-3p binding site was found in the 3′ UTR of the SP1 gene (Figure 3A). This was confirmed by dual-luciferase reporter assays. Luciferase activities of WT1-3′ UTR and WT2-3′ UTR in 293T cells transfected with miR-128-3p mimic exhibited a remarkable decrease, while those of mutant-type (MUT)1-3′ UTR and MUT2-3′ UTR co-transfected with miR-128-3p mimic were unaltered (Figure 3B, *P* < 0.05). Furthermore, no disparities were recognized after transfection with NC mimic (Figure 3B, *P* > 0.05). The above findings suggested miR-128-3p directly targeted and downregulated SP1.

### Intrathecal Administration of miR-128-3p Mimic and Inhibitor Strongly Regulates SP1 Expression *in vivo* After I/R

**FIGURE 4:**
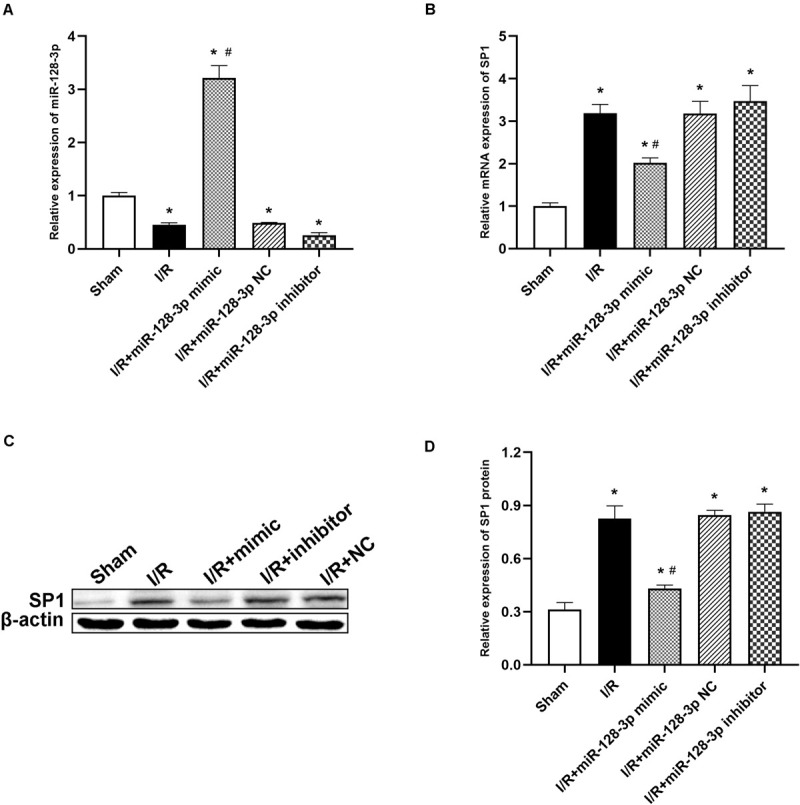
Effects of miR-128-3p mimic and inhibitor on miR-128-3p and SP1 levels in the L4–L6 segments of spinal cord specimens. **(A)** miR-128-3p expression levels in various groups. **(B)** Quantification of SP1 mRNA expression in various groups by qRT-PCR. **(C,D)** Western blot analysis of SP1 protein levels in various groups. β-actin was used as a loading control. One-way ANOVA was performed for the multiple- group followed by the Tukey’s test. *P < 0.05 vs. sham group, ^#^*P* < 0.05 vs. I/R and NC group.

To investigate miR-128-3p’s interaction with SP1 in the spinal cord tissue, miR-128-3p mimic, inhibitor and NC were administered by intrathecal injection for 3 days prior to ischemia. Then, qRT-PCR and Western blotting were carried out to assess miR-128-3p and SP1 mRNA and protein levels in the spinal cord, respectively. Compared with the I/R group, miR-128-3p was upregulated after administration of miR-128-3p mimic (Figure 4A *P* < 0.05) but showed no remarkable differences in the miR-128-3p NC group (*P* > 0.05). Significantly, pretreatment with miR-128-3p mimic remarkably reduced I/R-associated SP1 upregulation at the mRNA and protein levels at 12 h post-I/R; conversely, pretreatment with miR-128-3p inhibitor reversed the above effects (Figures 4B–D; *P* < 0.05). The above findings jointly suggested that the levels of SP1 were decreased due to overexpressed miR-128-3p and increased after miR-128-3p downregulation.

### Intrathecal Pretreatment With miR-128-3p Mimic or AV-sh-SP1 Ameliorates Neurological Function and Histologic Evaluation After I/R

**FIGURE 5:**
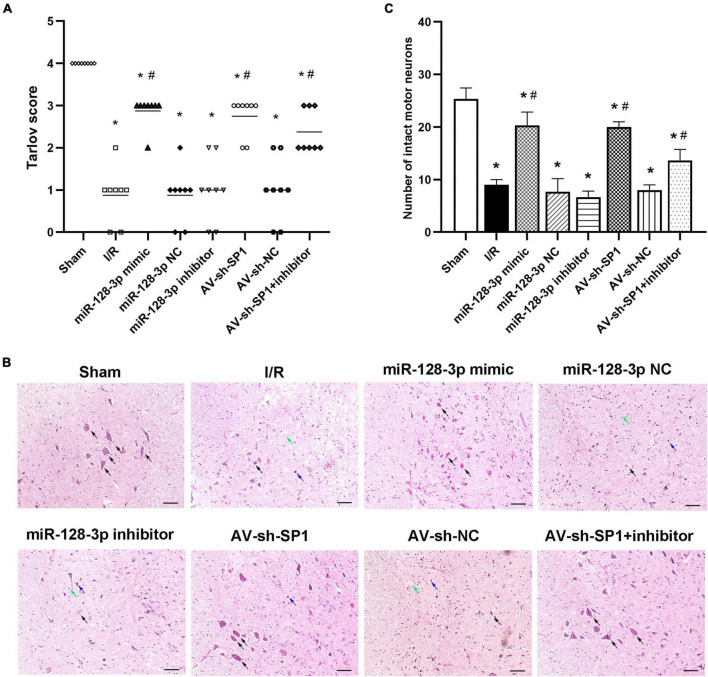
Effects of miR-128-3p mimic and AV-sh-SP1 on neurological function and histologic evaluation after I/R. **(A)** Neurological function scores at 12 h after I/R in eight groups. Tarlov scores ranged from 0 (paraplegia) to 4 (normal). Each symbol represents one rat (*n* = 8). **(B)** Representative sections of L4–L6 spinal cord segments in the central horn of gray matter stained with hematoxylin and eosin 12 h after I/R in eight groups. Scale bar = 100 μm. **(C)** Numbers of intact motor neurons of ventral gray matter in the eight groups. The black arrows indicate normal neurons. The blue arrows indicate dead neurons with a diffuse cytoplasm without cellar structure. The green arrows indicate loosened tissue organization. *P < 0.05 vs. sham group. ^#^*P* < 0.05 vs. IR or NC group.

Neurological function in the eight groups according to the Tarlov scores post-I/R was presented in Figure 5A Tarlov scores in the I/R group were significantly reduced at 12 h post-I/R compared with the sham group (*P* < 0.05). In comparison with the I/R and miR-128-3p NC groups, the miR-128-3p mimic group showed markedly elevated scores, whereas administration of miR-128-3p inhibitor resulted in lower Tarlov scores (*P* < 0.05). Tarlov scores were comparable in the I/R and miR-128-3p NC groups. Knockdown of SP1 using AV increased Tarlov scores (*P* < 0.05). Meanwhile, Tarlov scores were similar in the I/R and AV-sh-NC groups. Additionally, the miR-128-3p inhibitor + AV-sh-SP1 showed higher Tarlov scores (*P* < 0.05 vs. IR group) indicating AV-sh-SP1 could eliminate the damage caused by miR-128-3p inhibitor. Consequently, spinal cord I/R injury induced severe neurological deficits of lower limbs; meanwhile, miR-128-3p mimic and AV-sh-SP1 relieved neurological damage, while miR-128-3p inhibitor aggravated neurological deterioration. Likewise, histological findings at 12 h post-I/R revealed that the numbers of intact neurons in the anterior horn were significantly decreased, as shown by the existence of diffusely eosinophilic cytoplasm without cell structure, extensive vacuolation and pkynotic nuclei of neurons. Whereas, more intact neurons with basophilic substance and multipolar structure were seen after treatment with miR-128-3p mimic and AV-sh-SP1; miR-128-3p inhibitor increased necrotic or dead neurons in the anterior horn, while treatment with miR-128-3p inhibitor + AV-sh-SP1 reversed this effect (Figure 5B). The numbers of neurons with normal morphology in the anterior horn of spinal cord at 12 h post-I/R was significantly decreased in the I/R group (9 ± 1.000) than in the sham group (25.33 ± 2.082), miR-128-3p mimic group (20.33 ± 2.517), AV-sh-SP1 group (20 ± 1.000) and AV-sh-SP1 + inhibitor group (13.67 ± 2.082) (Figures 5C; *P* < 0.05). In the miR-128-3p NC, miR-128-3p inhibitor and AV-sh-NC group the numbers of neurons with normal morphology in the anterior horn were 7.667 ± 2.517, 6.667 ± 1.155, and 8 ± 1.000, respectively and had no marked differences compared with I/R group (*P* > 0.05).

### Intrathecal Pretreatment With miR-128-3p Mimic or AV-sh-SP1 Ameliorates BSCB Leakage and Proinflammatory Cytokine Release After I/R

**FIGURE 6:**
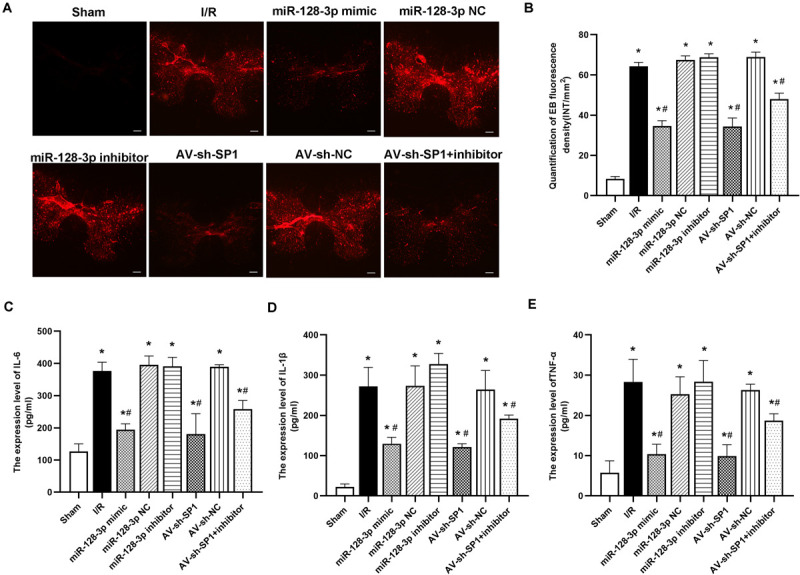
Effects of miR-128-3p mimic and AV-sh-SP1 on blood–spinal cord barrier (BSCB) integrity and IL-6, IL-1β and TNF-α amounts after I/R. **(A)** EB extravasation visualized as red fluorescence by microscopy to evaluate the alterations in BSCB permeability in the eight groups. Scale bar = 200 μm. **(B)** Quantification of EB fluorescence intensity (INT/mm^2^). **(C–E)** Measurements of IL-6, IL-1β, and TNF-α expression levels by ELISA in the eight groups. One-way ANOVA was performed for multiple-group comparisons, followed by the Tukey’s test. *P < 0.05 vs. sham group. ^#^*P* < 0.05 vs. IR or NC group.

I/R-induced BSCB leakage was evaluated by EB fluorescent dye as described in previous reports (Fang et al., 2013a; Li et al., 2014b,c). As shown in Figures 6A,B, I/R notably increased red fluorescence in the gray matter at 12 h after surgery in comparison with sham animals (*P* < 0.05). Intrathecal injection of miR-128-3p mimic or AV-sh-SP1 remarkably reduced fluorescence levels (*P* < 0.05), whereas miR-128-3p inhibitor aggravated BSCB leakage. Moreover, the degree and intensity of fluorescence in the miR-128-3p inhibitor + AV-sh-SP1 group were lower levels (*P* < 0.05). The I/R and NC groups also had no marked differences (*P* > 0.05).

Next, we assessed whether neuronal inflammation is an important response in spinal cord I/R injury development. As shown in Figures 6C–E, proinflammatory cytokines, including IL-6, TNF-α, and IL-1β, had remarkably increased amounts at 12 h post-I/R vs. sham group (*P* < 0.05). Interestingly, intrathecal injection of miR-128-3p mimic or AV-sh-SP1 reduced the release of IL-6, TNF-α and IL-1β (vs. I/R group; all *P* < 0.05). In contrast, miR-128-3p inhibitor increased IL-6, TNF-α, and IL-1β amounts (vs. I/R group; all *P* < 0.05), while, IL-6, TNF-α, and IL-1β amounts were reduced in the miR-128-3p inhibitor + AV-sh-SP1 group (*P* < 0.05 vs. inhibitor group), and were comparable in the I/R and miR-128-3P NC groups.

### Intrathecal Pretreatment With miR-128-3p Mimic or AV-sh-SP1 Ameliorates Neuronal Apoptosis After I/R

**FIGURE 7:**
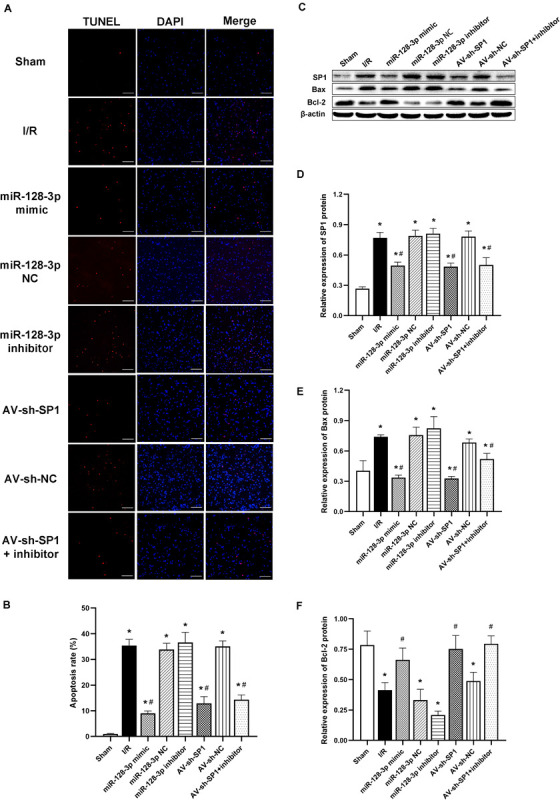
Effects of miR-128-3p mimic and AV-sh-SP1 on neuronal apoptosis and SP1, Bax and Bcl-2 amounts after I/R. **(A)** Cell apoptosis in the ventral gray matter by TUNEL staining (green)and DAPI (blue). Scale bar = 100 μm. **(B)** Quantification of TUNEL-positive neurons in the ventral horn averaged across three independent experiments. **(C–F)** Representative Western blotting and quantitative protein analyses of SP1, Bax and Bcl-2 in the various groups. One-way ANOVA was performed for multiple-group comparisons, followed by the Tukey’s test. *P < 0.05 vs. sham group. ^#^*P* < 0.05 vs. IR or NC group.

Apoptotic cells in spinal cord samples were represented by TUNEL-positive signals. The results in Figures 7A,B showed that the rate of apoptosis was markedly elevated in the I/R group in comparison with sham animals (*P* < 0.05). Apoptosis rate increase was partially restored in the miR-128-p mimic and AV-sh-SP1 group, respectively (both *P* < 0.05), whereas miR-128-3p inhibitor promoted apoptosis. Interestingly, apoptosis rate in the miR-128-3p inhibitor group was restored by AV-sh-SP1 (*P* < 0.05). In addition, the I/R and NC groups showed comparable values (*P* > 0.05). Similarly, to assess the possible mechanism underlying the effects of miR-128-3p and SP1 on apoptosis post-I/R, SP1, Bcl-2 and Bax amounts were assessed by Western blotting upon transfection with miR-128-3p mimic, inhibitor and AV-sh-SP1, respectively. As shown in Figures 7C–F, SP1 expression levels were significantly upregulated in the I/R group in comparison with sham group (*P* < 0.05). Meanwhile, miR-128-3p mimic or AV-sh-SP1 remarkably downregulated SP1 (*P* < 0.05). Conversely, miR-128-3p inhibitor upregulated SP1. Interestingly, in the miR-128-3p + AV-sh-SP1 group, the expression of SP1 was inhibited (*P* < 0.05). Bax expression levels changed with the same trends observed for SP1. Bcl-2 was markedly downregulated in the I/R group in comparison with sham animals (*P* < 0.05), and miR-128-3p mimic or AV-sh-SP1 promoted Bcl-2 expression (*P* < 0.05), which was suppressed by miR-128-3p inhibitor. After administration of miR-128-3p inhibitor + AV-sh-SP1, Bcl-2 protein amounts were also elevated (*P* > 0.05). The I/R and NC groups were also comparable. Hence, upregulation of miR-128-3p suppressed apoptosis in rat spinal cord injury after I/R by suppressing SP1 expression.

## Discussion

Prominent findings unveiled by this work are that elevated miR-128-3p amounts downregulated the transcription factor SP1, thus ameliorating the impairment of neurons induced by I/R via regulating inflammation and apoptosis.

Cumulative evidences illustrate that miRs are novel molecular targets modulating the pathogenesis of I/R injury (Li et al., 2015, 2017; Liu et al., 2017; Li et al., 2018; Li et al., 2020). The associations of miR-128-3p with central nervous system diseases have been assessed previously. For example, Krichevsky et al. (2006) suggested that miR-128-3p is upregulated in the development of the central nervous system. Additionally, high miR-128-3p amounts in neurons alleviate motor deficits in a mouse model of PD (Tan et al., 2013), and it was recently confirmed that miR-128-3p overexpression in the 1-methyl-4-pheny-1,2,3,6-tetrahydropyridine (MPTP)-injured mouse model of PD reduced hippocampal neuron apoptosis (Zhang et al., 2020). Meanwhile, miR-128-3p protected neurons from apoptosis at the early stage of cerebral ischemia (Mao et al., 2017). The above data suggest miR-128-3p’s regulatory role in neuroinflammation and neuroapoptosis post-injury. However, discrepant findings have been reported. While miR-128-3p is considered to enhance neuronal survival in ischemia-induced brain damage (Mao et al., 2017), its inhibition has been shown to induce the protection of cardiomyocytes from I/R injury (Chen et al., 2017). One miR can modulate many target genes, whereas one target gene could be modulated by multiple miRs (Brandenburger et al., 2012; Ksiazek-Winiarek et al., 2013), and functional target genes might depend on pathological conditions, treatments and the effector organ. Thus, it is very possible to encounter discrepancy in expression while examining the same miR in distinct conditions.

Given that neuroinflammation and neuroapoptosis have been shown to be critical phenotypes of spinal cord I/R injury (Li et al., 2015; Bao et al., 2018; Li et al., 2018), we further investigated whether miR-128-3p protected against spinal cord I/R injury by inhibiting inflammation and apoptosis. In this work, miR-128-3p was downregulated in the spinal cord at 6, 12, 24, 36, and 48 h, respectively, post-I/R in comparison with sham animals, and miR-128-3p content reached its lowest point at 12 h after I/R. We, therefore, selected 12 h post-I/R for subsequent assays. Consistently, distinct ameliorations in hind-limb motor function, preservation degrees of BSCB permeability, reduction in inflammatory factors in ELISA (IL-1β, TNF-α, and IL-6) and numbers of complete neurons were obtained after miR-128-3p mimic administration, as reflected by increased Tarlov scores and reduced EB extravasation. Moreover, we performed TUNEL staining and measured protein levels of Bax and Bcl-2, to investigate the mechanisms by which miR-128-3p might attenuate the neuronal apoptosis after I/R (Figures 7A,C). The results showed that TUNEL-positive cells mainly presented a neuronal morphology and were distributed in the anterior horn with increased Bax and decreased Bcl-2 in Western blotting. Following miR-128-3p mimic administration, the TUNEL-positive cells decreased in the anterior horn with decreased Bax and increased Bcl-2. Based on these data, we hypothesized that miR-128-3p might induce neuroprotective effects in spinal cord I/R injury by ameliorating inflammatory cytokine production and apoptotic rate.

To explore the mechanism underlying the neuroprotective effects inducted by miR-128-3p overexpression on spinal cord I/R injury, potential mRNA targeting was examined. MiRs require complementary binding with ≥6 consecutive nucleotides in the 3′ UTR of an mRNA target (Li et al., 2015). Based on TargetScanHuman 7.1 (Li et al., 2018). SP1 mRNA had 8 complementary nucleotides in the 3′ UTR, and the temporal expression patterns of SP1 and miR-128-3p exhibited a negative correlation at 12 h after reperfusion. In accordance with this result, reduced luciferase activity was found after co-transfection with a plasmid harboring the WT 3′ UTR of SP1 and miR-128-3p mimic. To avoid the cellular crosstalk and interactions *in vivo*, these effects were next assessed after pretreatment of rats with miR-128-3p mimic, control and inhibitor, respectively (Li et al., 2017). Administration of miR-128-3p mimic significantly reduced SP1 mRNA and protein amounts, while control and inhibitor miR showed no reductions, receptively (Figures 4B–D). Jointly, the above data suggest that miR-128-3p directly suppresses spinal cord SP1 following I/R.

SP1 is a frequently reported DNA-binding protein with a C2H2 zinc finger structure that controls gene transcription in multiple physiological and pathological processes (Chu, 2012). SP1 and related proteins contribute to multiple critical cellular events, including cell growth, differentiation, malignant transformation and apoptosis (Vizcaino et al., 2015). Based on previous literature, SP1 overexpression promotes apoptosis by itself, and malignant cells with SP1 overexpression can avoid SP1-associated apoptosis (Deniaud et al., 2006). In addition, some reports have found that SP1 was upregulated, and SP1 suppression exerted neuroprotection in experimental PD (Yao et al., 2018; Cai et al., 2020). SP1 also favors inflammation in many pathologies (Shin et al., 2015; Yang et al., 2018), and its absence reduces TNF-α, IL-1β, and IL-6 amounts in hypoxia-induced human umbilical vein endothelial cells (HUVECs) (Yang et al., 2018). These findings corroborate Qin et al. (2018), who revealed that suppressing the activation of SP1 effectively inhibits OGD/R-associated inflammation in microglia. Recently, emerging evidences indicate that expression of SP1 under ischemia/hypoxic conditions is increased (Eltzschig et al., 2009; Li et al., 2019). In addition, SP1 expression is upregulated following acute kidney damage associated with I/R (Chen et al., 2017). However, a report described anti-necroptosis effects for human-induced pluripotent stem cell-derived mesenchymal stromal cells-extracellular vesicles (hiPSC-MSCs-EVs) in renal I/R injury by delivering SP1 into target renal cells; meanwhile, SP1 knockout blunted renal protection by hiPSC-MSCs-EVs in rats with I/R injury (Yuan et al., 2017). Such discrepancy may be explained by distinct disease models and study time points for specific cell-type activation. A previous study also noted increased SP1 expression in the cortex after brain I/R damage and in primary neurons post-OGD/R (Sun et al., 2015), providing novel insights into the endogenous pathways that prevent ischemia-associated neuronal injury. Moreover, SP1 suppression delays neuronal loss and prolongs survival (Garcia-Morales et al., 2019). Additionally, in the current study, intrathecal injection of AV-sh-SP1 not only induced comparably lower levels of SP1 and resulted in poor production of IL-6, IL-1β, TNF-α, and Bax, but also upregulated Bcl-2, improved neurological function and histological findings, dwindled TUNEL-positive cells in anterior horn, and attenuated EB leakage, in comparison with the I/R group. The current findings suggest that SP1 might represent an inflammation and apoptosis-associated transcription factor, which promotes neuronal survival in spinal cord I/R injury. Moreover, we demonstrated that miR-128-3p targeted SP1, corroborating previous findings that miR-128 plays an essential role in glioma progression via SP1 suppression (Dong et al., 2014). Another report confirmed that high miR-128-3p expression suppresses cell proliferation and differentiation in bovine skeletal muscle satellite cells via SP1 downregulation (Dai et al., 2016).

## Conclusion

Overall, miR-128-3p suppression provides remarkable protection of the spinal cord I/R injury, involving both anti-inflammatory and anti-apoptotic effects. Prophylactic approaches for spinal cord protection are essential, and this is the first report demonstrating that the miR-128-3p/SP1 axis has critical functions in neuroprotection upon spinal cord ischemia. This report further confirms that miRNAs are neuroprotective molecules affecting spinal cord I/R injury.

## Data Availability Statement

The raw data supporting the conclusions of this article will be made available by the authors, without undue reservation, to any qualified researcher.

## Ethics Statement

The animal study was reviewed and approved by Animal Ethics Review Committee for Animal Experimentation of China Medical University.

## Author Contributions

DW, FC, and HM designed and performed the experiments and obtained the data. DW, BF, ZZ, and YD performed the statistical analysis and wrote the sections of the manuscript. XT wrote the first draft of the manuscript. All authors contributed to manuscript revision, read, and approved the submitted version.

## Conflict of Interest

The authors declare that the research was conducted in the absence of any commercial or financial relationships that could be construed as a potential conflict of interest.

## Footnotes

1
http://www.targetscan.org

2
https://imagej.nih.gov/ij/

## Abbreviations

ANOVA: analysis of variance
AV: adenovirus
BCL-2: B cell lymphoma 2
AR: apoptotic rate
BAX: BCL-2 associated X
BCA: bicinchoninic acid
BSCB: blood–spinal cord barrier
DAPI: 4′6-diamidino-2-phenylindole
DMEM: Dulbecco’s Modified Eagle Medium
ECL: chemiluminescence
EB: Evans blue
ELISA: Enzyme-linked immunosorbent Assay
IL: interleukin
I/R: ischemia/reperfusion
miRs: microRNAs
HE: hematoxylin and eosin
i.v.: intravenous injection
i.p.: intraperitoneal injection
MPTP: 1-methyl-4-phenyl-1,2,3,6-tetrahydropyridine
MUT: mutant-type
NC: negative control
NS: normal saline
OGD/R: oxygen-glucose deprivation/reperfusion
PD: Parkinson’s disease
PVDF: polyvinylidene fluoride
PMSF: phenylmethylsulfonyl fluoride
qRT-PCR: quantitative real-time polymerase chain reaction
RIPA: radio immunoprecipitation
SP1: specificity protein 1
SDS-PAGE: sodium dodecyl sulfate-polyacrylamide gel electrophoresis
TNF: tumor necrosis factor
TMR: tetramethy-rhodamine
TUNEL: terminal deoxynucleotidyl transferase dUTP nick end labeling
UTR: untranslated region
WT: wild-type.
