# Corrigendum: MiR-128-3p Alleviates Spinal Cord Ischemia/Reperfusion Injury Associated Neuroinflammation and Cellular Apoptosis *via* SP1 Suppression in Rat

**DOI:** 10.3389/fnins.2021.707766

**Published:** 2022-01-14

**Authors:** Dan Wang, Fengshou Chen, Bo Fang, Zaili Zhang, Yan Dong, Xiangyi Tong, Hong Ma

**Affiliations:** Department of Anesthesiology, The First Hospital of China Medical University, Shenyang, China

**Keywords:** spinal cord ischemia/reperfusion injury, miR-128-3p, SP1, neuroinflammation, apoptosis, rat

In the original article, in Figure 5B, the same image was used for the sixth image (**AV-sh-SP1**) and the eighth image (**AV-sh-SP1+** **inhibitor**) by mistake. During the final submission of figures for this manuscript's publication, the eighth image was inadvertently replaced by the sixth image. In the history of our submitted files, the corrected version of Figure 5 is the version we originally submitted, as shown below.

**Figure 5:**
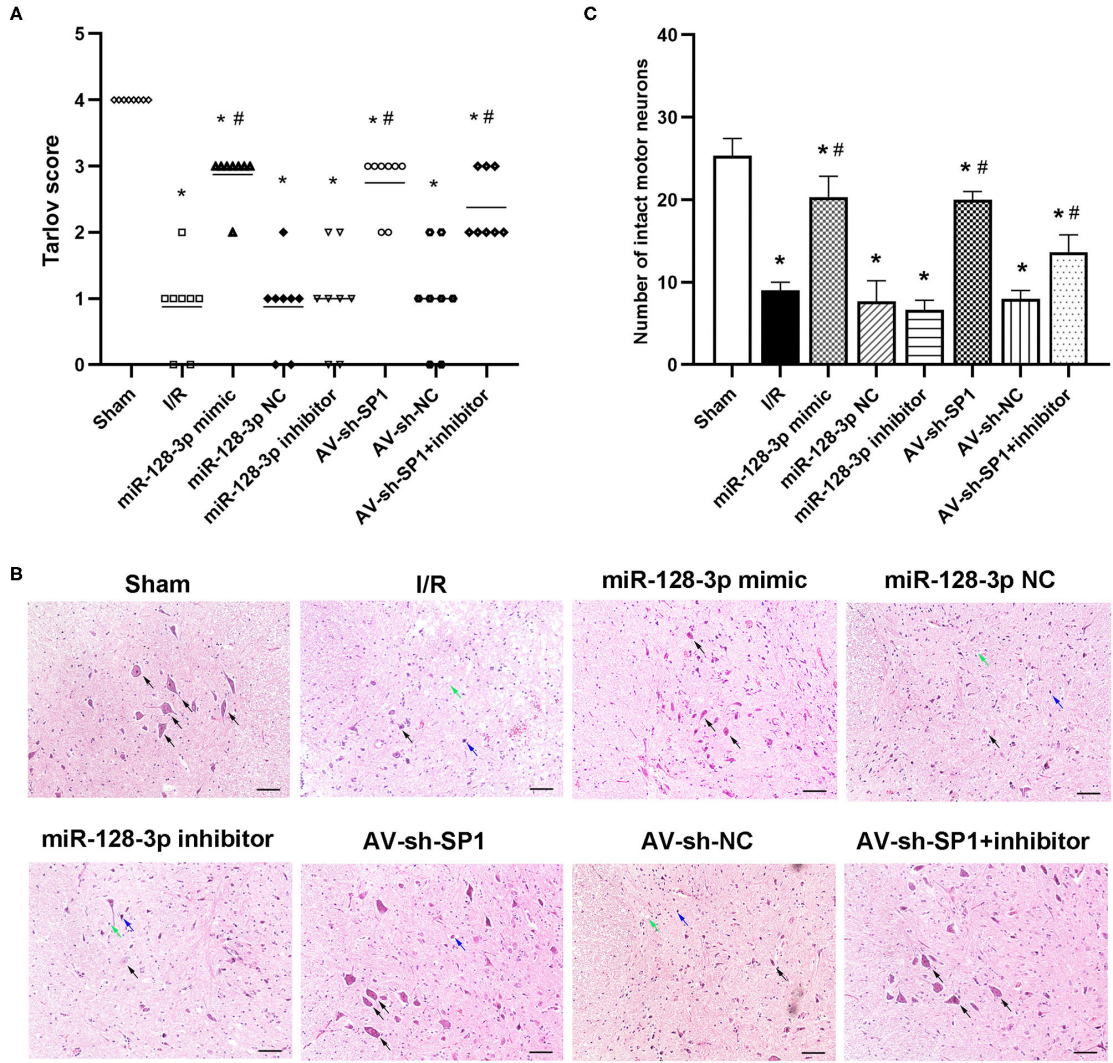
Effects of miR-128-3p mimic and AV-sh-SP1 on neurological function and histologic evaluation after I/R. **(A)** Neurological function scores at 12 h after I/R in eight groups. Tarlov scores ranged from 0 (paraplegia) to 4 (normal). Each symbol represents one rat (*n* = 8). **(B)** Representative sections of L4–L6 spinal cord segments in the central horn of gray matter stained with hematoxylin and eosin 12 h after I/R in eight groups. Scale bar = 100 μm. **(C)** Numbers of intact motor neurons of ventral gray matter in the eight groups. The black arrows indicate normal neurons. The blue arrows indicate dead neurons with a diffuse cytoplasm without cellar structure. The green arrows indicate loosened tissue organization. ^*^P < 0.05 vs. sham group. ^#^*P* < 0.05 vs. IR or NC group.

The authors apologize for this error and state that this does not change the scientific conclusions of the article in any way. The original article has been updated.

## Publisher's Note

All claims expressed in this article are solely those of the authors and do not necessarily represent those of their affiliated organizations, or those of the publisher, the editors and the reviewers. Any product that may be evaluated in this article, or claim that may be made by its manufacturer, is not guaranteed or endorsed by the publisher.

